# TDP-43 Regulation of AChE Expression Can Mediate ALS-Like Phenotype in Zebrafish

**DOI:** 10.3390/cells10020221

**Published:** 2021-01-22

**Authors:** Maria-Letizia Campanari, Anca Marian, Sorana Ciura, Edor Kabashi

**Affiliations:** Team “Translational Research for Neuronlogical Diseases”, Institut Imagine Inserm U1163, Université de Paris; Sorbonne Université, Université Pierre et Marie Curie (UPMC), Université de Paris 06, Unité Mixte 75, Institut National de la Santé et de la Recherche Médicale (INSERM) Unité 1127, Centre National de la Recherche Scientifique (CNRS) Unité Mixte de Recherche 7225 Institut du Cerveau et de la Moelle Épinière (ICM), 75013 Paris, France; anca.marian@institutimagine.org (A.M.); sorana.ciura@institutimagine.org (S.C.); edor.kabashi@institutimagine.org (E.K.)

**Keywords:** amyotrophic lateral sclerosis (ALS), TAR DNA-binding protein 43 (TDP-43), neuromuscular junction (NMJ), knockdown (KD), acetylcholinesterase (AChE)

## Abstract

The “distal axonopathy” hypothesis in amyotrophic lateral sclerosis (ALS) proposes that pathological changes occur at the neuromuscular junction (NMJ) early in the disease. While acetylcholinesterase (AChE) plays an important role in the functionality of the NMJ, its potential role in ALS remains unexplored. Here, we identified AChE as a limiting factor regulating muscle/motor neuron connection in a vertebrate model of ALS. Knockdown of the TAR DNA-binding protein 43 (TDP-43) orthologue in zebrafish resulted in early defects of motor functions coupled with NMJ disassembly. We found that a partially depleted tdp-43 caused a decrease of ache expression. Importantly, human AChE overexpression reduced the phenotypic defects in the tdp-43 loss of function model, with amelioration of post- and pre-synaptic deficits at the NMJ. In conclusion, our results provide a better understanding of the role of TDP-43 in the NMJ organization and indicate AChE as a contributing factor in the pathology of ALS. In particular, it may be implicated in the early defects that characterize NMJs in this major neurodegenerative disorder.

## 1. Introduction

Amyotrophic lateral sclerosis (ALS) is a multifactorial, multisystem disease caused by progressive loss of both upper and lower motor neurons [1]. The pathological signature of ALS is the presence of TAR DNA-binding protein 43 (TDP-43) inclusion bodies, the protein from the gene *TARDBP* [2], in the affected regions of brain and spinal cord in over 95% of affected patients with its consequent clearance from the nucleus [3,4]. The discovery of causative *TARDBP* mutations in 2008 [5,6] brought this gene to the forefront of neurodegeneration research, not only as a major pathological marker but also as a causative factor in sporadic and familial ALS. TDP-43 is an heterogeneous nuclear ribonucleoprotein (hnRNP) [7,8] able to bind both nucleic acids and proteins, involved in a wide range of RNA processes, including transcription, transport, stability, and splicing [9]. Together with additional RNA-binding proteins linked to ALS (reviewed in [10]), TDP-43 gives RNA metabolism a central role in ALS pathogeny. Therefore, among the hundreds of TDP-43′s targets, the determination of a relevant factor for ALS development is crucial.

Increasing evidence shows early morphological and biochemical changes at the neuromuscular junction (NMJ) before complete muscle fiber denervation and functional symptom onset in ALS patients [11,12]. In particular, in mutant SOD1^G93A^ mouse, which replicates several key features of ALS, reduced NMJ complexity at presymptomatic stages has been described, corresponding to an augmented number of disassembled acetylcholine (ACh) receptor clusters on muscle from the mutant SOD1 transgenic mice [13].

Structurally, the NMJ is a highly complex cholinergic synapse where the majority of the molecules responsible for its formation are also crucial for its stability and function at the adult stage [14].

In this context, the enzyme acetylcholinesterase (AChE) is a master regulator of the signal transduction. In mammals, AChE exists as three distinct variants coming from alternative exon splicing (AChE T, R, and H) [15,16,17]. The main specie, the T (tail), is recruited by specific anchor proteins and targeted to the plasma membrane (if produced by neurons [18]) or to NMJ basal lamina (if produced by muscles [19]), where it terminates neurotransmission by hydrolyzing the neurotransmitter ACh [15]. AChE activity regulates synaptic ACh levels that, in turn, guarantee the functionality and the integrity of the synapse itself [15,20,21,22,23].

In addition, the enzyme has several non-classical roles in neuronal regeneration and development, as demonstrated in dopaminergic neurons [24,25]; in rat cultured dorsal root ganglion neurons [26]; and in amphibian, avian, and mammalian glia and neurons [27,28]. NMJ impairments have been demonstrated in a mouse knockout (KO) for AChE [21], as well as in a mouse model specifically lacking of AChE anchored at the NMJ [29]. Moreover, transgenic mice overexpressing human AChE display pathological changes at the NMJs [30,31]. Altogether, AChE level and activity play an important role in NMJ viability.

In mammals, our understanding of AChE cellular mechanisms is complicated due to the presence of butyrylcholinesterase (BChE), another enzyme able to hydrolyze ACh, although less effectively [32], and through alternative splicing, which is sensible to AChE inhibitors [33]. Zebrafish (zf; *Danio rerio*) permit the overcoming of these issues since this vertebrate does not appear to express a functional bche and has not been reported to undergo alternative splicing at the respective 5′ or 3′ ends [34]. Thus, it only presents a T variant, providing the opportunity to specifically investigate the ache function at the plasma membrane and during NMJ development and maintenance during vertebrate embryogenesis in vivo. As previously reported, the inactive form of ache induces progressive motility defects and severe reduction of ach receptor clusters in two independent transgenic zf lines [35,36], consistent with a role for ache in synapse stability.

Several studies on pathological tissues [37,38,39] and transgenic mouse models [40] have proposed a potential role for AChE in ALS. Very recently, this role has been supported by using a spatial transcriptomic analysis approach that showed a reduced expression of AChE transcript in spinal cord regions primarily related to ALS symptoms onset [41].

Data summary: In this study, we describe for the first time a functional link between tdp-43 and ache in zebrafish. For this purpose, we employed the zf model previously described by our team [42], where knockdown of tdp-43 leads to symptoms reminiscent of ALS disease, such as disruption of axonal projections from spinal motor neurons and reduced locomotion. In this model, ache expression is reduced then restored with human TDP-43 overexpression. Importantly, the overexpression of the human AChE-T form partially rescues the motor and NMJ deficits seen in tdp-43 loss of function. Altogether, these results confirm an important role that ACHE through the functional interaction TDP-43 could play in ALS pathogenesis and open new avenues into our understanding of this neuromuscular disorder.

## 2. Materials and Methods

### 2.1. Zebrafish Studies

Adult and larval zebrafish (*Danio rerio*) were maintained at the ICM (Institute du Cerveau et de la Moelle épinière, Paris, France) fish facilities and bred according to the National and European Guidelines for Animal Welfare. Experiments were performed on wild-type embryos from AB and TL genetic strains. Zebrafish stored in incubators at 28.5 degrees remain at embryonic stages until approximately the end of the third day post-fertilization, before they hatch out of the chorion [43,44]. All procedures were approved by the Institutional Ethics Committee at the Research Center of the ICM.

### 2.2. Microinjections

A morpholino, binding to the ATG sequence, responsible for translational initiation, was designed to knockdown (KD) tardbp (AMO-TDP-43; 5′-GTACATCTCGGCCATCTTTCCTCAG-3′) and AChE gene (AMO-AChE; 5′-CTGAGGTCTTCATGGCTTCTTTTCA-3′). A control morpholino (mis-std), containing mismatch nucleotides and not binding anywhere in the zebrafish genome, was used to assess the specificity of the observed phenotype (5′-CCTCTTACCTCAgTTACAATTTATA-3′). Human TARDBP wild-type (WT) mRNA (WT-TDP-43) was transcribed from NotI-linearized pCS2þ using SP6 polymerase with the mMESSAGE Machine Kit (Ambion, Thermo Fisher Scientific, Illkirch-Graffenstaden, France). AChE-T plasmid (WT-AChET) under the cytomegalovirus (CMV) promoter–enhancer was a generous gift from Dr. J. Saez Valero (Instituto de Neurociencias, Alicante, Spain) [45]. Microinjections were performed at 0.6 mM for AMO-TDP-43, 0.45 mM for AMO-AChE mM, and 150 ng/uL for WT-AChET. Low doses were chosen to minimize morpholino-induced developmental delay and toxicity, as well as to yield a consistent phenotype. Injections were performed in 1–4 cell stage blastulae. Embryos were maintained at 28 °C and manually dechorionated using fine forceps at 24 hpf. After behavioral test, 15 to 20 fish were deyolked and solubilized in ice-cold extraction buffer: 50 mM Tris-HCl (pH 7.4)/500 mM NaCl/5 mM Ethylenediaminetetraacetic acid EDTA/1% (*w*/*v*) Nonidet P−40/0.5% (*w*/*v*) Triton X-100 supplemented with a cocktail of protease inhibitors (Complete Protease Inhibitor Cocktail, Roche, Mannheim, Germany). Fish were then sonicated and centrifuged at 14,000× *g* at 4 °C for 20 min. Supernatants were collected and frozen at −80 °C until biochemical analysis.

### 2.3. Touch-Evoked Escape Response (TEER)

Zebrafish embryos at 48 hpf were analyzed to identify any major morphological abnormalities (body/head malformations and size). Subsequently, embryos that did not display any developmental abnormalities were touched lightly at the level of the tail with a pipette tip in order to evaluate their locomotor behavior. TEER episodes were performed only in zebrafish that appeared morphologically normal and were recorded with a Grasshopper 2 Camera (Point Grey Research, Richmond, British Columbia, Canada) at 30 Hz. The videos were then analyzed using the manual tracking plugin of ImageJ 1.45r software as previously described [46].

### 2.4. Immunofluorescence

Animals were fixed in 4% paraformaldehyde for 3 h at room temperature. After fixation, the embryos were rinsed several times with PBS and then incubated in PBS containing 1 mg/mL collagenase (20 min, C9891, Sigma-Aldrich, Saint-Quentin Fallavier, France) to remove skin. The collagenase was washed off with PBS Triton X-100 (PBST; 1 h) and heads were cut away. After an incubation of 30 min in blocking solution (1% BSA, 1% triton, PBS, 2% goat serum), the embryos were incubated overnight at 4 °C in synaptic vesicle 2 (sv2, 1:200; Developmental Studies Hybridoma Bank; University of Iowa, Iowa USA) antibody diluted in blocking solution. The embryos were then washed and incubated for 30 min in PBST containing α-bungarotoxin conjugated to Alexa 488 (αBTX, 1:1000; Abcam). The embryos were rinsed several times with PBST and then incubated in freshly prepared block solution containing a secondary antibody (Alexa Fluor 568, 1:1000; Life Technologies, Saint-Aubin, France) for 3h at RT before mounting on glass slide in 50% glycerol. The NMJs were visualized using a Leica SP8 Inverted scanning confocal microscope. All captured images of stained embryos were processed using Imaris Image Analysis software and ImageJ.

### 2.5. Staining for Acetylcholinesterase Activity

The Karnovsky and Roots method adapted for zebrafish was used [36]. Optimal cutting temperature compound (OCT)-embedded embryos were cut at the cryostat (20 µm section wide). Slices were incubated for 2h at RT in thiocholine substrate (5 mg) in 0.1 M sodium hydrogen maleate, 0.1 M sodium citrate, 30 mM CuSO_4_, and 5 mM potassium ferricyanide (pH 6.0). The reaction was stopped with washes in PBS. Then, the sections were dehydrated, cleared with xylene, coverslipped, and imaged with the Nanozoomer scanner (Hamamatsu, Massy, France).

### 2.6. AChE Enzyme Assay and Protein Determination

A modified micro assay version of the colorimetric Ellman’s method was used to measure AChE [47]. A total of 1 mU of AChE activity was defined as the number of nanomoles of acetylthiocholine hydrolyzed per minute at 22 °C. Total protein concentrations were determined using the BCA Protein Assay Kit (Thermo Scientific, Rockford, IL, USA).

### 2.7. RNA Isolation and Analysis of Transcripts by qPCR

Total RNA was isolated from injected fish using TRIzol Reagent (Sigma) according to the manufacturer’s protocol. First-strand cDNAs were obtained by reverse transcription of 1 μg of total RNA using the High-Capacity cDNA Reverse Transcription Kit (Roche), according to the manufacturer’s instructions. Quantitative PCR amplification was performed with SyBer2X Gene Expression Assays using the primers listed in Table S1. Data were analyzed transforming raw Cq values into relative quantification data using the delta Cq method.

### 2.8. Statistical Analysis

All data values for the zebrafish and cells experiments are expressed as mean with error bars representing standard errors of mean (SEM) with significance determined using one-way ANOVAs. Differences between groups were identified via Bonferroni post hoc comparisons. All analyses were performed using Prism 5.0 (Graph Pad, San Diego, CA, USA). Significance level was set at *p* < 0.05.

## 3. Results

### 3.1. TDP-43 Knockdown Induced Defective NMJ Structures in Zebrafish

**Figure 1:**
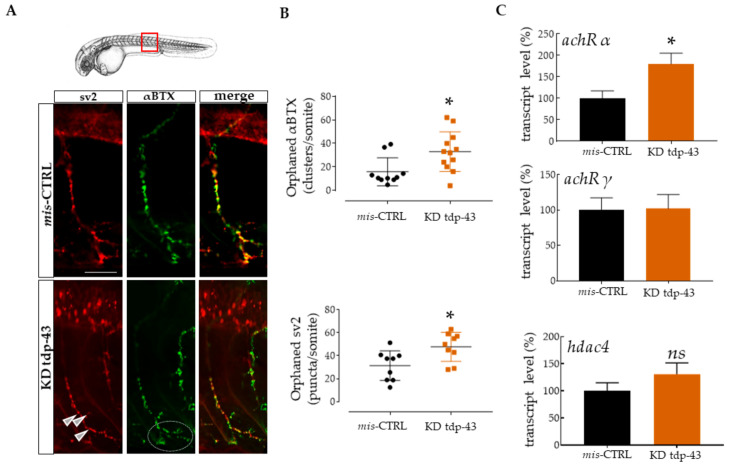
Knockdown (KD) of TAR DNA-binding protein 43 (tdp-43) caused motor impairments and defective neuromuscular junction (NMJ) structure. (**A**) Representative images of one ventral root projection double labeled for sv2 (presynaptic marker, red) and αBTX (postsynaptic, green). mis-CTRL embryos showed extensive red and green colocalization when converted in spots by Imaris software, as can be seen from the yellow staining seen in the merged images. Scale bar equals 10 μm. (**B**) Quantification of orphaned αBTX clusters for somite and of orphaned sv2 pre-synaptic marker. These clusters described as “spots” were measured following 3D reconstruction of the ventral CaP axon extension by Imaris [52]. Each point in these graphs represents the number of orphan spots per axon extension. Six fish were used for this study. (**C**) Relative transcript levels of *ach receptors α* and *γ,* and muscle histone deacetylase 4 *(hdac4)* expressed as a percentage and normalized to tubulin. Error bars represent standard error of the mean (SEM). * *p* < 0.05. ns = not significant.

Several genes mutated in ALS patients and implicated in motor neuron degeneration have been studied in zebrafish, including superoxide dismutase (SOD1) [48], alsin (ALS2) [49], the elongated protein 3 (ELP3), FUS [50], and TDP-43 [42]. The overexpression of the mutated form these genes in zebrafish leads to short motor axons extension and premature branching associated with deficient swimming behavior in response to touch. To better characterize the defects associated to TDP-43 loss of function, we performed double-immunostaining for synaptic vesicle 2 (sv2; a presynaptic marker) and alpha bungarotoxin (αBTX; a toxin that binds ach receptors irreversibly) in a zebrafish model previously described [42] where embryos injected with tdp-43 morpholino (KD tdp-43) display shorter swim duration, distance, and swim velocity when compared to control (mis-CTRL) (Figure S1). Then, the plugin “Spot” by Imaris software permitted the identification and the quantification of pre- and post-synaptic contacts [51,52]. In mis-CTRL, normal NMJs were characterized by the close juxtaposition of pre- and post-synaptic components (Figure 1A). In particular, the αBTX staining showed ach receptor clusters regularly disposed along the muscle fiber. On the contrary, upon reduction of tdp-43 expression, the number of orphaned (denervated) BTX clusters and sv2 puncta increased when compared with control (αBTX orphans: 33 ± 4.88, *n* = 12 for tdp-43 KD, vs. 15.82 ± 3.786, *n* = 10 for mis-CTRL, *p* < 0.05; sv2 orphans: 47.67 ± 4.206, *n* = 9 for tdp-43 KD, vs. 31.41 ± 4.311, *n* = 9 for mis-CTRL, *p* < 0.05; Figure 1B). Interestingly, these aberrant NMJ connections were accompanied by ach receptor cluster fragmentation on muscle membrane (Figure 1A, white circle).

Signs of endplate denervation in ALS have been observed in patients [53,54] as well as in murine models of ALS [14].

In order to test a possible phenomenon of denervation, we used quantitative PCR to determine the relative transcript expression levels for zebrafish ach receptor subunits α (*achRα*) and γ (*achR γ*), and for the muscle histone deacetylase 4 (*hdac4*), already described as molecular markers of denervation intensity [55,56,57]. In our model, *achR*α was increased (180.4 ± 24.64, *n* = 11 for tdp-43 KD, vs. 100 ± 17.21, *n* = 16 for mis-CTRL, *p* < 0.05; Figure 1C, upper panel), and *hdac4* expression was slightly increased, although it was not significant (Figure 1C, bottom). Contrarily, the fetal subunit *achR* γ was not changed (Figure 1C, middle panel).

### 3.2. TDP-43 LoF Caused Decreased Ache Expression

**Figure 2:**
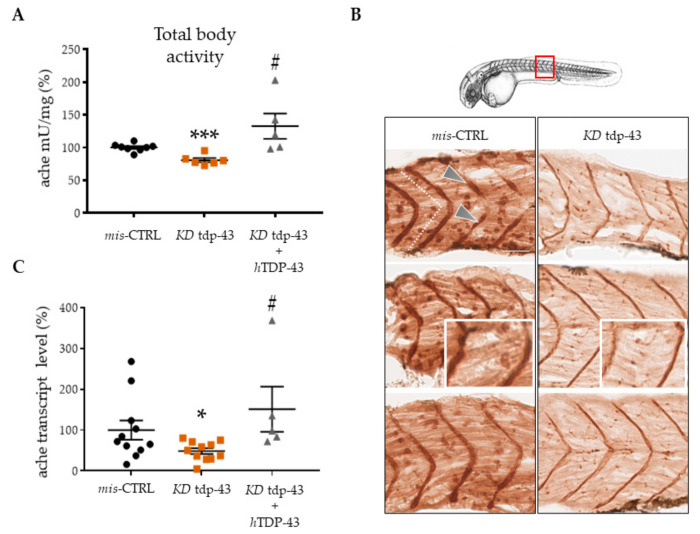
TDP-43 loss of function (LoF) caused decreased acetylcholinesterase (ache) expression. (**A**) Quantitation of ache activity by the spectrophotometric method of Ellman in extracts prepared from 48 hpf embryos. (**B**) ache activity revealed by Karnovsky and Roots staining in sagittal sections of mis-CTRL and tdp-43 KD embryos. Pictures represent the magnification of the 10th somite (red square). After tdp-43 KD, the intensity of immunostaining was weaker, with spots representing functional NMJ units appearing as smaller and diffuse. Scale bar equals 10 μm. (**C**) Messenger RNA levels of the ache transcript were measured by qPCR from zebrafish (zf) extracts. Values were expressed in percentage and normalized to gapdh. Error bars represent standard error of the mean (SEM). * *p* < 0.05 difference with control. *** *p* < 0.001 difference with control. # *p* < 0.05 difference between KD tdp-43′ and KD tdp-43+hTDP-43.

Since TDP-43 is known to control the fate of several transcripts involved in synaptic formation and transmission, we determined whether TDP-43 could regulate AChE levels. Current knowledge on NMJ formation point on the significance of AChE activity in its stabilization [14]. For this reason, we determined ache activity by Ellman assay spectrophotometry on total fish protein extract (Figure 2A). TDP-43 KD embryos present an important reduction of ache activity (80.78 ± 3.186, *n* = 6 for tdp-43 KD, vs. 100.1 ± 2.126, *n* = 8 for mis-CTRL, *p* < 0.001) that can be rescued by the expression of the corresponding human gene (hTDP-43) (127.1 ± 21.82, *n* = 5 for tdp-43 KD + hTDP-43, vs. 100.1 ± 2.126, *n* = 8 for mis-CTRL). Because of the impossibility to extract proteins specifically from muscle, we used Karnovsky and Roots reaction on sagittal embryo sections to detect ache activity in muscles (Figure 2B). In mis-CTRL embryos, the motor endplates, where the membrane-bound ache is active, appeared as brown dots, particularly enriched in a vertical band in the center of each somite (white dashed lines). These puncta represented the NMJ established by fast muscle fibers. Brown precipitation was also detected in the vertical myosepta (arrows), where NMJ for slow muscle are present [58].

Compared to mis-CTRL, ache activity intensity was found to be significantly decreased in tdp-43 deficient embryos. Interestingly, brown spots looked smaller and more diffuse (insert).

Furthermore, we performed qPCR analysis in order to quantify ache expression at the transcript level. After KD of tdp-43, endogenous ache transcript was found to be reduced by 40% (48.52 ± 7.067, *n* = 11 for tdp-43 KD, vs. 99.99 ± 23.44, *n* = 11 for mis-CTRL, *p* < 0.05; Figure 2C). Moreover, ache transcript expression was rescued when tdp-43 activity was re-established, thus confirming the importance of tdp-43 in the maintenance of ache levels.

### 3.3. Functional Interactions between Ache and Tardbp

To determine whether a functional interaction occurs between ache and tdp-43, we performed rescue experiments by co-injecting the human *AChE-T* variant (hAChE-T) or *TARDBP* (hTDP-43) genes after KD of either of these genes in zebrafish.

**Figure 3:**
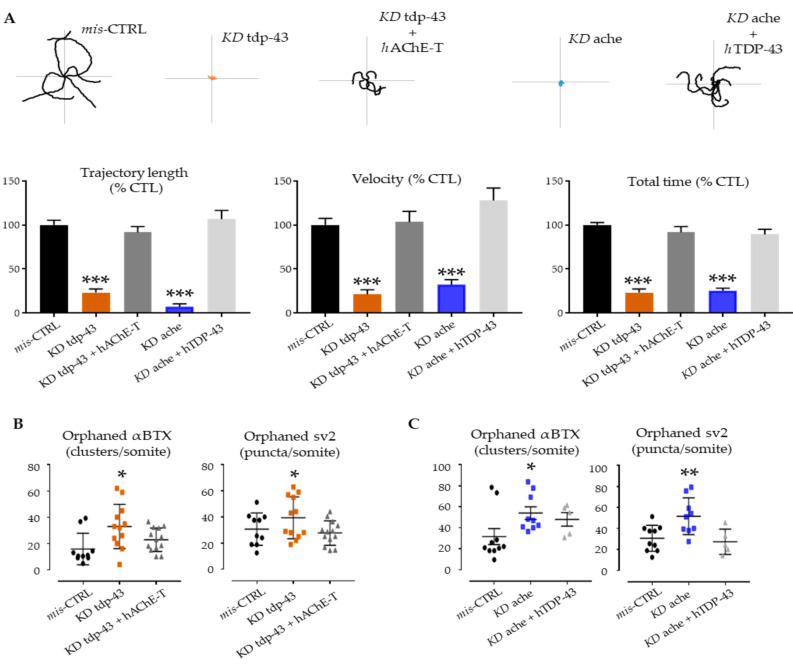
Genetic interactions between *ache* and *tardbp*. (**A**) Examples of locomotor path traces from single- and double-injected groups. tdp-43 rescued ache KD and human ACHE overexpression reduced phenotypic features observed upon tdp-43 KD. Column graph represents the quantification of motor parameters measured at the TEER test: trajectory length, swimming velocity, and total time. (**B**,**C**) At the NMJ level, the functional rescue was accompanied with amelioration of post- and pre-synaptic deficits. After injection with human TDP-43 and AChE-T, the KD phenotypes were ameliorated with a general decrease of orphan spots indicating amelioration in the formation of proper NMJ units. Error bars represent standard error of the mean (SEM). * *p* < 0.05, ** *p* < 0.01, *** *p* < 0.001 difference with control.

Expression of hAChE-T in tdp-43 knockdown fish (orange) significantly improved the swimming behavior at 48hpf (trajectory length: 94.49 ± 11.97, *n* = 27 for tdp-43 KD + hAChE-T, vs. *7 ± 3.613, n* = 12 for tdp-43 KD vs. 100 ± 10.19, *n* = 26 for mis-CTRL; velocity: 91.65 ± 6.572, *n* = 27 for tdp-43 KD + hAChE-T, vs. 23.27 ± 4.229, *n* = 12 for tdp-43 KD vs. 100 ± 3.924, *n* = 26 for mis-CTRL; total time: 103.4 ± 12.09, *n* = 27 for tdp-43 KD + hAChE-T, vs. *21.43 ± 5.14, n* = 12 for tdp-43 KD vs. 100 ± 10.62, *n* = 26 for mis-CTRL; Figure 3A, grey columns). Indeed, the trajectory of these morphants was found to be significantly longer when compared with tdp-43 AMO-injected fish.

As previously described, KD ache embryos (blue) gradually lost motility up to 48 hpf [35]. The over-expression of hTDP-43 in ache AMO-injected fish ameliorated the swimming trajectory performances measured at the TEER assay (trajectory length: 106.7 ± 9.842, *n* = 50 for ache KD + hTDP-43, vs. 100 ± 10.19, *n* = 26 for mis-CTRL; velocity: 127.8 ± 14.16, *n* = 50 for ache KD + hTDP-43, vs. 100 ± 10.62, *n* = 26 for mis-CTRL; total time: 89.17 ± 5.973, *n* = 50 for ache KD + hTDP-43, vs. 100 ± 3.924, *n* = 26 for mis-CTRL; Figure 3A, light grey columns).

Since tdp-43 knockdown in zebrafish induces ach receptors reorganization, reminiscent of the diffuse achRs distribution previously seen in mutant zebrafish (achesb55) lacking of ache activity [35], we evaluated the NMJ staining after rescue experiments.

In both double injections (KD tdp-43 + hAChE-T and KD ache + hTDP-43), we did not detect any pre/post synaptic orphans as compared to tdp-43 or ache KD conditions (Figure 3B,C). Furthermore, α-bungarotoxin labeling overlapped with sv2 staining, and motor axon morphology was comparable to mis-CTRL embryos (Figure S2).

These data suggest the existence of a functional relationship where ache and tardbp operate and confirm the important role that ache has during development and NMJ stability.

## 4. Discussion

In this study, we extended our understanding of a major pathological factor in ALS, TDP-43, and its potential contribution in this neuromuscular disorder. Here, we established that tdp-43 plays an important action in regulating ache expression and levels in zebrafish. We demonstrated that ache levels that remain membrane-bound are decreased upon tdp-43 knockdown. Importantly, motor phenotype and the NMJ’s defects due to the tdp-43 depletion were rescued upon restoration of ache levels and activity.

In the overwhelming majority of ALS cases, TDP-43-mediated neurodegeneration is primarily caused by the loss of normal function of TDP-43 in nuclei without being mutated with a consequent accumulation in the cytosol resulting in pathological aggregation [9,59,60]. However, the shift to a pathological state is still unclear, especially in muscle fibers, where TDP-43 aggregates remain to be unraveled [61]. In this tissue, cytosolic TDP-43 has been shown to assemble in oligomers, where it recruits specific RNA and proteins directly involved in skeletal muscle formation and regeneration [62]. The increased assembly or decreased clearance of this pool could be the source of TDP-43-aggregates that commonly occur in neuromuscular disease [62,63]. In general, there is a growing acceptance of a pivotal role of TDP-43 in muscle. Indeed, TDP-43 depletion leads to locomotive and NMJs anatomical defects in *Drosophila* [64] as well as in zebrafish [42]. Our findings strongly support this role and put in evidence an NMJ remodeling partially dependent on the TDP-43/AChE pathway.

The relevance of these findings is evident when taking into consideration the role that AChE displays during neuronal development and NMJ synapse stabilization independently of its activity in cholinergic neurotransmission [65]. Thus, prior to synapse formation in vertebrates, AChE is expressed in both immature postmitotic neuroblasts that are about to extend the first long tracts and myotomal target cells, reviewed in [66]. 

Despite AChE being considered an “old actor” [67] in scientific research, the role of this factor in axonal growth and maintenance of the nerve-muscle system is not completely understood. Several in vitro studies have shown that ACh influences neurite extension by inhibiting neurite outgrowth [68,69,70,71]. Thus, a possible role for embryonic AChE would be to neutralize ACh inhibition and create a corridor for neurite growth. Another explanation comes from AChE sequence homology with Neuroligin 1, a membrane cell-adhesion protein of the family of neuroligins [72], that binds a specific set of neurexins, another family of cell surface proteins [73], regulating neuronal axogenesis independently of synaptic activity [74]. Therefore, it has been demonstrated in vitro that AChE can compete with Neuroligin 1 for the extracellular binding to the common ligand Neurexin, thus regulating neuritogenesis [75] and synaptogenesis [76]. Therefore, an AChE–neurexin recognition in vivo for inter-neuronal recognition and axon pathfinding should not be excluded.

In the mature NMJ instead, the AChE classical role involves the hydrolysis of ACh [67], and has been shown to be critical for NMJ stability [20,21,22,23,77]. AChE activity inhibition in fact induces ACh accumulation in the synaptic cleft, which in turn causes (1) the repeated activation of the nicotinic acetylcholine receptors (AChRs) and their consequent desensitization [20]; (2) the decrease of AChRs number at synapses (as compensatory mechanism) [21]; and (3) the fragmentation of AChRs clusters, since ACh elicits a negative effect on postsynaptic apparatus stabilization [22,23]. Indeed, NMJs are fragmented in mouse model lacking a functional AChE [21,29].

Similarly, the zebrafish model lacking ache catalytic activity presents smaller and fragmented ach receptors clusters, reminiscent of the anatomical abnormalities observed in muscle/NMJ biopsies from ALS patients [53,78]. Moreover, the *achR* α subunits are increased and *hdac4* expression augmented. The increase of these factors is particularly interesting since they are clearly upregulated in ALS-denervated muscle [54,56,79,80] and are considered markers of muscle atrophy. However, we cannot exclude the presence of re-innervating motorneurons, as NMJs in these embryos are smaller and aberrant branching of motorneurons is observed. Even though γ subunit levels are not altered to properly understand the implication of tdp-43 in denervation/reinnervation mechanisms, the levels of this factor should be measured at further time points of larval development.

In our tdp-43 KD model, NMJs clearly lose their integrity, and ache down-expression can be one of the major causes of NMJ dysfunctions. The discovery that tdp-43 LoF decreases ache expression in zebrafish could provide a novel mechanistic insight for nerve-muscle integrity disruption in ALS pathogenesis. Since TDP-43 downregulation appears to be an early defect in ALS, AChE expression could also be altered at early pre-symptomatic stages of the disease, leading to NMJ remodeling. Indeed, to reinforce these findings, a recent study that performed spatial transcriptomic analysis in ALS pathological tissue observed reduced AChE levels [41]. Since Neuroligin 1 was also shown to be a target of TDP-43 regulation in mouse brain, these factors could participate in parallel to regulate the neurogenesis and axogenesis at the level of NMJ [81]. Therefore, future studies are warranted to establish whether TDP-43 binds and regulates the transcription of *AChE* mRNA or rather indirectly TDP-43 and AChE can exert a role in NMJ maintenance through trans-acting factors [82].

Certain limitations arise from the usage of zebrafish models in this current study. Importantly, compared with other vertebrates, zebrafish embryos appear to be devoid of ache splicing [34]. For this reason, it will be important to eventually confirm and extend these findings using a transgenic mouse model of ALS, as well as on induced pluripotent stem cell (iPSC)-derived neurons and muscle co-cultures obtained from sporadic and familiar ALS patients. Together with these in vivo results, these approaches could also provide powerful tools to test this hypothesis and eventually to validate compounds that are capable of boosting AChE expression at the NMJ as potential therapeutic avenues in ALS and related neuromuscular disorders. Overall, our work gives novel insight into TDP-43 molecular mechanisms and supports the hypothesis of a direct involvement of AChE in ALS NMJ remodeling.

## Data Availability

Data is contained within the article or supplementary material.

## Supplementary Materials

The following are available online at https://www.mdpi.com/2073-4409/10/2/221/s1, Figure S1: Trajectory and TEER measurements after TDP-43 knock-down, Figure S2: Immunostaining for SV2 and αBTX was used to evaluate NMJs organization, Table S1: Primers list.
