# Supplementary material for: TDP-43 Regulation of AChE Expression Can Mediate ALS-Like Phenotype in Zebrafish
Source: Cells. 2021 Jan 22;10(2):221. doi: 10.3390/cells10020221 (PMC7911940; doi:10.3390/cells10020221)
Supplement: Supplementary file 1 [file cells-10-00221-s001.zip › Supplementari Table 1.docx]

| **Gene** | **Sequence** |
| --- | --- |
| **Gapdh** | Qiagen (QT02095786) |
| **AChE** | Qiagen (QT00209069) |
| **achRα-F** | TCGTCCTGTTAGCCATTTTA |
| **achRα-R** | GATTAACTTCATCCACGCTG |
| **hdac4-F** | GATTATCTGGCAGCATTCAG |
| **hdac4-R** | GAAAACTCATTCGCTATCGG |
| **achRγ-F** | GACACCTGAGCAGGAGGAAG |
| **achRγ-R** | GGACACACACTGCTGCACTT |

**Supplementary Table 1.** Primers list.
