# Supplementary figures and images for: TDP-43 Regulation of AChE Expression Can Mediate ALS-Like Phenotype in Zebrafish
Source: Cells. 2021 Jan 22;10(2):221. doi: 10.3390/cells10020221 (PMC7911940; doi:10.3390/cells10020221)

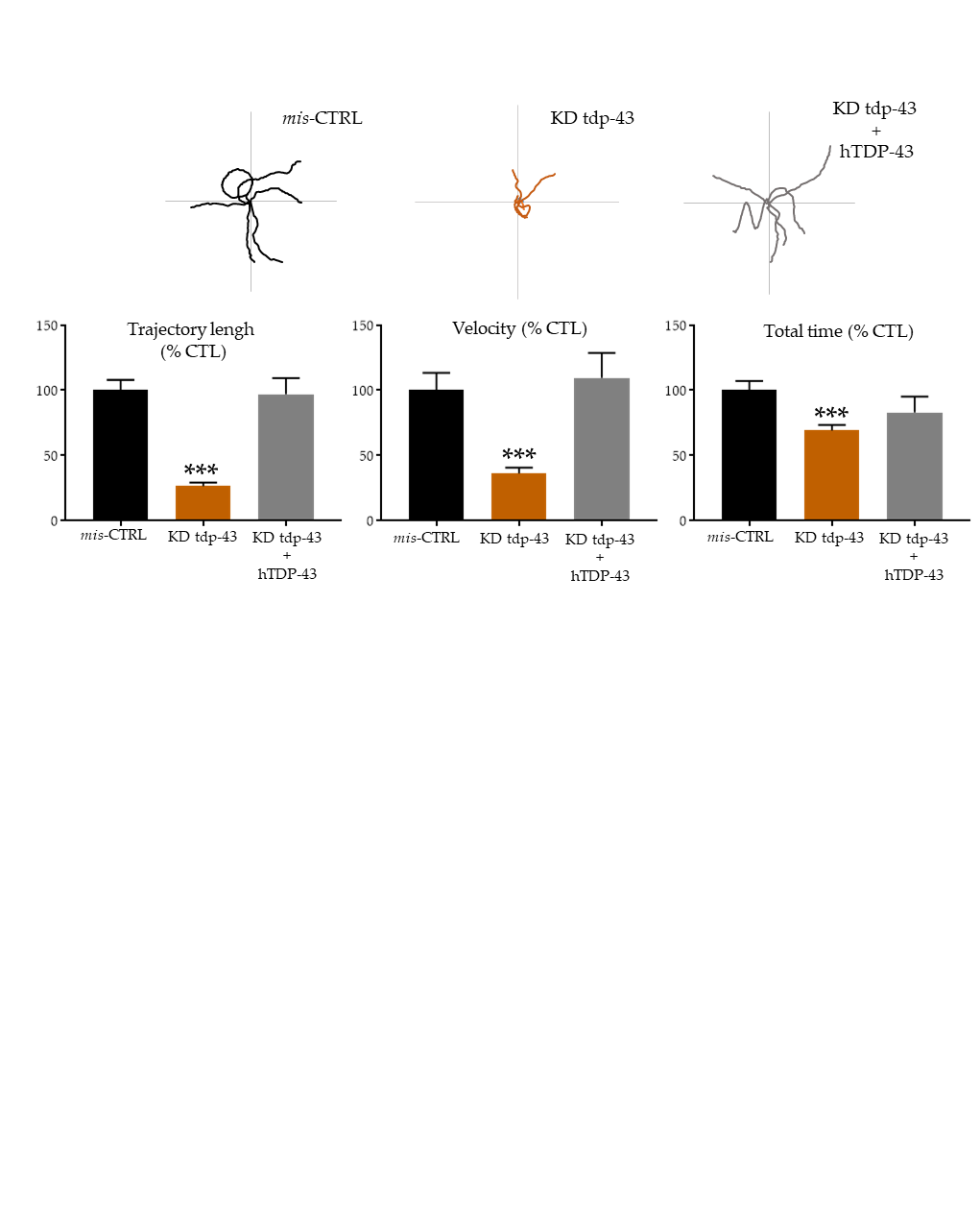

Supplement: Supplementary file 1 [file cells-10-00221-s001.zip › Supplementary Figure 1.tif]

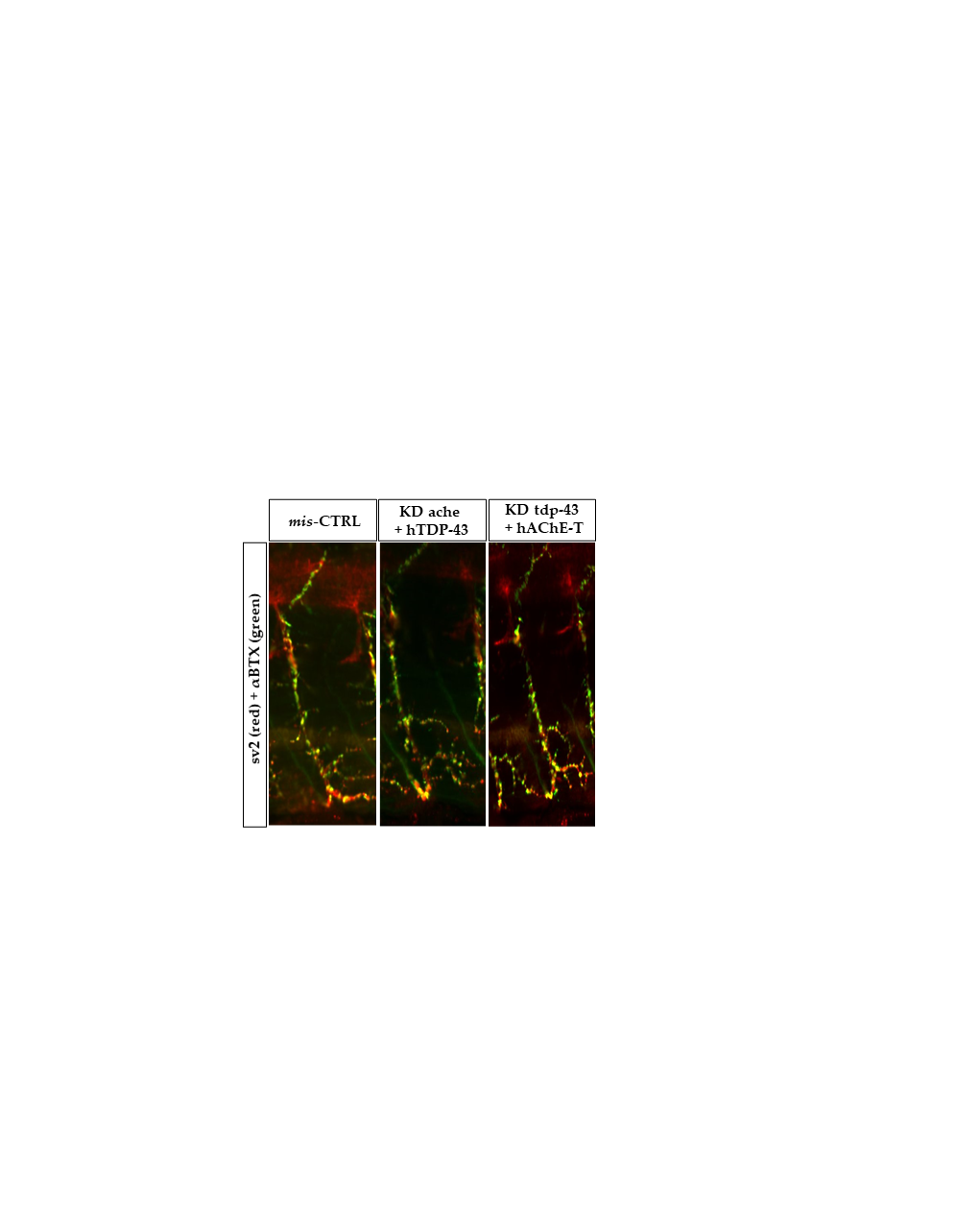

Supplement: Supplementary file 1 [file cells-10-00221-s001.zip › Supplementary Figure 2.tif]
